# Supervised consumption site enables cost savings by avoiding emergency services: a cost analysis study

**DOI:** 10.1186/s12954-022-00609-5

**Published:** 2022-03-28

**Authors:** Shahreen Khair, Cathy A. Eastwood, Mingshan Lu, Jennifer Jackson

**Affiliations:** 1 Department of Community Health Sciences, Cumming School of Medicine, University of Calgary, TRW Building, 3280 Hospital Dr NW, Calgary, AB T2N 4Z6 Canada; 2 Community Health Sciences, Centre for Health Informatics, Cumming School of Medicine, University of Calgary, TRW Building, 3280 Hospital Dr NW, Calgary, AB T2N 4Z6 Canada; 3 Department of Economics, The University of Calgary, 2500 University Drive NW, Calgary, AB T2N 1N4 Canada; 4 Faculty of Nursing, University of Calgary, Professional Faculties Building, 2500 University Drive NW, Calgary, AB T2N 1N4 Canada

**Keywords:** Supervised consumption services, Opioids, Overdose, Emergency services, Cost analysis

## Abstract

**Background and aims:**

We report on a cost analysis study, using population level data to determine the emergency service costs avoided from emergency overdose management at supervised consumption services (SCS).

**Design:**

We completed a cost analysis from a payer’s perspective. In this setting, there is a single-payer model of service delivery.

**Setting:**

In Calgary, Canada, ‘Safeworks Harm Reduction Program’ was established in late 2017 and offers 24/7 access to SCS. The facility is a nurse-led service, available for client drop-in. We conducted a cost analysis for the entire duration of the program from November 2017 to January 2020, a period of 2 years and 3 months.

**Methods:**

We assessed costs using the following factors from government health databases: monthly operational costs of providing services for drug consumption, cost of providing ambulance pre-hospital care for clients with overdoses who could not be revived at the facility, cost of initial treatment in an emergency department, and benefit of costs averted from overdoses that were successfully managed at the SCS.

**Results:**

The proportion of clients who have overdosed at the SCS has decreased steadily for the duration of the program. The number of overdoses that can be managed on site at the SCS has trended upward, currently 98%. Each overdose that is managed at the SCS produces approximately $1600 CAD in cost savings, with a savings of over $2.3 million for the lifetime of the program.

**Conclusion:**

Overdose management at an SCS creates cost savings by offsetting costs required for managing overdoses using emergency department and pre-hospital ambulance services.

**Supplementary Information:**

The online version contains supplementary material available at 10.1186/s12954-022-00609-5.

## Main text

### Introduction

Supervised consumption services (SCS) are a harm reduction intervention to provide a sanctioned area for clients to use substances, under medical supervision, with a legal exemption [1]. A primary aim of SCS is overdose prevention and early intervention for overdose management. SCS are widely accepted as decreasing fatal accidental overdoses [2]. SCS in Canada began with the creation of Insite in Vancouver in 2003 [3]. SCSs aimed to prevent overdoses and decrease the use of conventional services for overdose response, including emergency medical services and emergency departments.

There is substantial evidence of the benefits of SCS. Insite, in Vancouver, has been widely studied and demonstrated social and economic benefits across a range of measures [2–7]. The cost savings relating to human immunodeficiency viruses (HIV) infections alone were enough to offset Insite’s operating costs [3, 6]. Estimates related to the prevention of Hepatitis C transmission also showed evidence of significant cost savings [3, 5]. Proposals for SCS illustrated projected cost effectiveness in Toronto [8], Victoria [9], Ottawa [8, 10], San Francisco [11] and Baltimore [12].

While there are well-documented benefits of SCS, studies are limited to a few sites [7]. Cost analyses have tended to focus on HIV prevention or economic benefit of prevented deaths, without including other aspects of the SCS programs [7]. There are challenges in evaluating costs. Clients at SCS may not be required to provide personal health numbers or identifying information, as the services are intended to be anonymous for client safety. However, SCS do collect population level data about service use. Our cost analysis study overcomes these limitations by evaluating the population level benefit of decreased use of emergency services, at an SCS site that has not been previously reported in the literature. In this article, we use population level data in a cost analysis, to determine costs avoided via emergency overdose management at SCS. Please note that all currencies are reported in Canadian dollars. At time of writing, $1 CAD = £0.59 GBP.

## Methods

Overdose management was the point of analysis in this study. Overdose (OD) management in this study is defined as the application of a medical intervention to a client who is not rousable, following consumption of an illicit substance. This includes application of oxygen, administration of naloxone, and/or calling an ambulance for emergency medical care. Verbally rousing the client, or clients with a ‘heavy nod’ were excluded from definitions of overdose in this study. Our definition of overdose was limited to overdoses requiring medical intervention that could be confirmed in clinical documentation. Oxygen is considered a medical intervention, because the primary cause of overdose-related death is hypoxia [13]. Overdoses occurring in the community are not included in this study.

### Research site

In Calgary, a large city in Canada, ‘Safeworks Harm Reduction Program’ was established in late 2017 and offers 24/7 access to SCS [14]. The facility is a nurse-led service, available for client drop-in. Registered Nurses are always available to reverse an overdose or resuscitate a client. The nurses are equipped to administer oxygen and/or naloxone, if needed. We conducted a cost analysis for the entire duration of the program. This covers 2 years and 3 months since the service began.

### Study design

This cost analysis was completed from a payer’s perspective. A payer in this study refers to the entity (provincial government) providing funding to run the service. The following factors were chosen based on the availability of data to evaluate the costs avoided due to SCSs: monthly operational costs of providing service for drug consumption, cost of providing ambulance care for clients with overdoses who could not be revived at the facility, costs of emergency department care, and benefit of ambulance and hospital costs averted from overdoses that were successfully managed at the SCS.

### Data

Overdose data were extracted from the monthly, publicly available Government opioid reports [15–17] for the months of November 2017 to January 2020. Six data points were imputed for the number of people who were provided oxygen and/or naloxone between June and November 2019, and the methodology is provided in Additional file 1.

### Analytic methods

We computed descriptive statistics for the dataset. In addition, we examined the trend in the number of clients who overdosed at the SCS site. To develop an accurate visualization of these data, we have calculated a percentage for the number of overdoses for each month per number of total visits for drug consumption to the SCS, illustrating an overall trend line for overdose rates.

### Costs

The costs were divided into two categories: (1) operating costs for providing services at the SCS site and (2) cost of ambulance and emergency department care for clients who overdosed at the SCS site but required additional intervention.

#### Operating costs of safe consumption services

The first component of the analysis comprised of the operating costs at the SCS site, at a per visit rate. A recent study [14] by the government of Alberta reports that the average cost per visit for drug consumption is $52. The operating costs include costs of administering oxygen and/or naloxone, the nurses’ wages, and equipment costs for providing oxygen and/or naloxone. The operating cost at the SCS was calculated by employing the following formula:

$$O_{{\text{C}}} = N_{{{\text{DC}}}} \;\left( {\$ 52} \right)$$

where *O*_C_ is the total operating cost and *N*_DC_ is the total number visits to the site for drug consumption each year. This calculation provided the total yearly cost at this site.

#### Cost of emergency medical services for overdoses

The second part of this analysis was the cost of emergency medical services (EMS_C_) for clients who could not be revived at the SCS site after an overdose and included both pre-hospital ambulance care and initial emergency department care. This cost was calculated by employing the following formula:

$${\text{EMS}}_{{\text{C}}} = \left\{ {\left( {T_{{{\text{OD}}}} } \right)\;\left( {\$ 385} \right)} \right\} + \left\{ {\left( {T_{{{\text{OD}}}} } \right)\;\left( {\$ 1061 + \$ 176} \right)} \right\}$$

where EMS_C_ is the total cost of clients needing emergency medical services. *T*_OD_ is the total number of clients who overdosed and were taken to emergency services for each year. The transport cost of utilizing an ambulance is $385 [18] per use. The cost for an emergency department visit for residents of Alberta, Canada, for overdose treatment is $ 1061. This number is based on using the Comprehensive Ambulatory Classification System [19] grouping methodology resource intensity weight (RIW) and cost of standard hospital stay from the Alberta Health Services (AHS) to calculate an average cost of an emergency visit due to an overdose. Specific codes for RIW were B184 (Poisoning without Intervention), B204 (Trauma High Resource Intervention), B217 (Trauma with Acute Admission/Transfer without High Resource Intervention), B224 (Trauma with Moderate Intervention) and B234 (Poisoning with Minor Intervention).

The cost of physician fees per emergency visit lasting 45 min is $176 [20] and is also factored into the cost of an emergency department visit for an overdose in Alberta. We recognize that additional costs would be incurred if clients needed admission to hospital or critical care. The frequency of these admissions was not available as population level data and, thus, was beyond the scope of this study.

### Benefits

Calgary’s SCS site has not experienced a single overdose-related death since its inception, as reported by AHS [14]. Therefore, we assume that ambulance and emergency department costs are averted due to efficient handling of overdoses at the SCS.

#### Benefit from avoiding emergency medical services for overdoses in the SCS

The benefits of managing overdoses at the SCS, without ambulance and emergency department care are calculated by the formula:

$${\text{EMSA}}_{{\text{B}}} = \left\{ {\left( {T_{{{\text{ODA}}}} } \right)\;\left( {\$ 385} \right)} \right\} + \left\{ {\left( {T_{{{\text{ODA}}}} } \right)\;\left( {\$ 1061 + \$ 176} \right)} \right\}$$

where EMSA_B_ is the total benefit from overdose-related emergency medical services averted that would require ambulance and physician costs. *T*_ODA_ is the total number of overdoses averted for each year.

#### Total benefit and net savings

We added the values for costs related to overdose ambulance and emergency department care that were averted to determine the total benefits of overdose management at the SCS.

## Results

Table 1 shows descriptive statistics that describe volume and treatments of the SCS in Calgary. Variables include the number of clients, proportion of visits used for drug consumption and referrals for other social services, total number of overdoses, and other related outcomes.

**Table 1:** Drug consumption site summary statistics at an SCS site in Calgary, Canada

Population outcomes	2017*^1^		2018		2019		2020*^2^	
	*n*	(%)	*n*	(%)	*n*	(%)	*n*	(%)
Number of unique clients	540		7970		12,645		1270	
Total number of visits	2551	(100)	51,791	(100)	69,028	(100)	6623	(100)
Visits used for drug consumption (*N*_DC_)	2108	(83)	42,386	(82)	58,629	(85)	5994	(91)
Visits for referrals to other services	70	(3)	329	(1)	1969	(3)	153	(2)
Total overdoses (ODs)	55	(100)	716	(100)	727	(100)	47	(100)
ODs provided oxygen	35	(64)	543	(76)	714	(98)	46	(98)
ODs provided naloxone	20	(36)	338	(47)	312	(43)	13	(28)
OD EMS (*T*_OD_)	6	(11)	51	(7)	29	(4)	1	(2)
OD EMS averted (*T*_ODA_)	49	(89)	665	(93)	698	(96)	46	(98)

2017*^1^, November and December data

2020*^2^, January data

The proportion of visits for drug consumption has increased over time, but it is notable that nearly 10% of clients who have accessed SCS between 2017 and 2020 are not doing so for drug consumption. This finding reinforces the role of the SCS in providing ancillary services, including referrals and wound care. The overall population for drug use at the SCS has increased, while the need for ambulance responses to overdoses has decreased over time. Presently, 98% of overdoses are managed on site. This rate has increased steadily since the opening of the SCS site. In the most recent full year of operation (2019), 698 overdoses were managed at the SCS site, thus avoiding ambulance calls and emergency department visits.

### Overdose trends

**Fig. 1:**
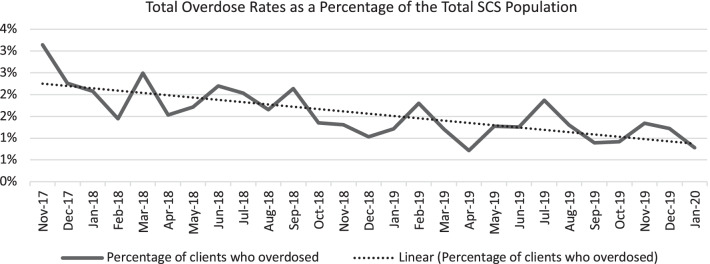
Trend analysis of overdoses over the period of supervised consumption site utilization. 2017*^1^, November and December data; 2020*^2^, January data

These data show that the number of overdoses at the site has been increasing over time. However, the number of visits for drug consumption has also increased over time. The linear trend line in Fig. 1 shows that the percentage of overdoses declined between November 2017 to January 2020.

### Costs

Since the inception of the SCS, service use volume has increased. The total costs, which includes the cost of operating the SCS site and the cost of emergency medical services for overdoses, ranged from $59,674 to $313,310 per month for November 2017 and January 2020. The annual operating cost for the most recent full year of operation (2019) was estimated at $3,048,708. Table 2 provides the total annual and monthly costs. SCS operating costs have increased over time, which reflects a corresponding increase in service use.

**Table 2:** SCS operating cost and cost of EMS for overdoses at SCS

Year	SCS operating costs	Cost of OD emergency medical services	Average total cost (monthly)	Total cost (annual)
2017*^1^	$109,616	$9732	$59,674	$119,348
2018	$2,204,072	$82,722	$190,566	$2,286,794
2019	$3,048,708	$47,038	$257,979	$3,095,746
2020*^2^	$311,688	$1622	$313,310	$313,310

2017*^1^, November and December data

2020*^2^, January data

### Benefits

#### Benefit from avoiding emergency medical services for overdoses in the SCS

Each overdose that is managed at the SCS produced a benefit of $1622 for January 2020 (Table 3). The benefit of averting the cost of ambulance and emergency department care ranges between $39,739 and $74,612 per month, from November 2017 to January 2020. Table 3 provides the total annual and monthly benefits.

**Table 3:** Benefit from avoiding emergency medical services for overdoses at the SCS

Year	Average emergency medical services benefits (monthly)	Emergency medical services benefits (annual)
2017*^1^	$39,739	$79,478
2018	$89,886	$1,078,630
2019	$94,346	$1,132,156
2020*^2^	$74,612	$74,612

2017*^1^, November and December data

2020*^2^, January data

Overall, there were $2,364,876 cost savings produced from the overdoses that were managed at the SCS site (Table 3), by avoiding the need for ambulance and emergency department services, over the life of the program to date. These costs use the minimum billing fee for the payer and exclude overdose-related hospitalization costs and, thus, likely underestimate total costs saved.

## Discussion

This study focuses on a harm reduction service, in the context of a drug poisoning crisis. Our study demonstrates that SCS can be justified in part by their benefits in avoiding costs related to managing overdoses. The declining trend of overdoses may indicate the effectiveness of the program in preventing overdoses, as well as managing them. A reduction in the number of overdoses would result in lowering the rate of overdose-related deaths and saving more lives, which adds value to the economy and society. The overdose reduction could be related to increased staff comfort with managing overdoses, client education around overdose prevention, fewer people overdosing and dying alone, or broader factors like the reduced toxicity of the drug supply. Future research could investigate these factors in more depth.

The cost savings of overdose management at the SCS, although substantial, were not sufficient to offset the operating cost of the program. However, this study examined only one aspect of the SCS’s potential benefits. Several authors found significant cost savings associated with reduced needle sharing at SCS [3, 4]. It is likely that the total cost of this SCS could be offset if additional variables were examined. There is potential for further analysis in future studies.

We predict there are secondary benefits, as our study demonstrated that the SCS prevents approximately 700 calls for ambulance services per year. This number is due to the 700 overdoses that are managed at the SCS, avoiding the need for ambulance and emergency department intervention. In addition to the cost savings, it is reasonable to project that other people requiring urgent care in Calgary may be able to access ambulance or emergency department care more quickly, with fewer overdose-related calls for these services. It has long been known that wait times are a primary determinant of patient satisfaction in emergency departments [21]. The SCS in this study prevented notable numbers of patients attending emergency departments each year, which is especially important in the context of COVID-19, where health services are under considerable strain.

Additionally, the SCS also provide other health  services, such as referrals for housing or drug treatment programs. There may be economic and social benefits from clients receiving such additional services, which they may have difficulty accessing elsewhere. Future studies can evaluate the cost benefits of SCS staff supporting clients to access other supportive services.

The methodology employed in this study may be applied to evaluate SCS sites at other locations. Overdose management has been considered in prospective cost analyses [8–12] but we have not found other published examples of this analysis with operating SCS.

Our study was conducted using available data since the inception of the SCS in late 2017. A small amount of data was imputed with no indication of underreporting of other data. Due to the limitations of the anonymous service in this study, more in-depth data on hospitalization rates and other social service referrals could not be obtained. Future studies could explore other ways to measure cost–benefit while respecting clients’ preferences to access the service anonymously. Emergency department cost estimates would be enhanced if centers had access to severity scores, variable costs over time, and information on patients who leave against medical advice. There are also opportunities to consider additional metrics, such as brain injuries that were avoided by rapid overdose treatment at an SCS site.

This study only includes an analysis of known overdoses at the SCS site. Future research could also examine the rates of overdoses in the community, to achieve a broader understanding of overdose management and prevention across settings.

The study does not employ an economic evaluation methodology due to time and data restrictions. The future of this SCS site is uncertain in its current political context. As a result, the scope and underlying methodologies are brief and allow limited analysis. Moreover, due to the COVID-19 pandemic, movements are restricted, rendering it infeasible to conduct primary surveys. There is also the potential for additional analysis of overdose scenarios occurring outside the SCS and resulting in overdose-related deaths, which could be calculated if these data were available. This analysis may strengthen the cost savings argument further.

Our study demonstrates the need to conduct economic evaluations of SCS relating to overdose management. The literature on SCS widely documents the direct benefits of these services, including reduced HIV and Hepatitis C infections, skin and soft-tissue infections, the economic benefits of preventing overdose-related deaths and the indirect benefits of social service referrals. Adding overdose management to future studies of the direct economic benefits of SCS will strengthen the analysis.

## Conclusion

In this study, we identified notable cost savings produced through overdose management at SCS. This reduced the reliance on ambulance and emergency department services. In addition, the number of overdoses at the SCS has decreased. The SCS thus offer direct savings, and secondary benefits from fewer visits to emergency departments. Future studies could explore these benefits in more depth.

## Supplementary Information

**Additional file 1.** Methodology for imputed data points.

## Data Availability

The datasets used and/or analyzed during the current study are available from the corresponding author on reasonable request.

## Abbreviations

SCS: Supervised consumption services
HIV: Human immunodeficiency viruses
OD: Overdose
EMS: Emergency Medical Services
RIW: Resource intensity weight
AHS: Alberta Health Services
