# Supplementary material for: Supervised consumption site enables cost savings by avoiding emergency services: a cost analysis study
Source: Harm Reduct J. 2022 Mar 28;19:32. doi: 10.1186/s12954-022-00609-5 (PMC8959556; doi:10.1186/s12954-022-00609-5)
Supplement: Supplementary file 1 — Additional file 1. Methodology for imputed data points. [file 12954_2022_609_MOESM1_ESM.pdf]

Appendix A

1 Problem definition

In the SCS dataset, data is missing for 2 columns, OD oxygen and OD Naloxone, from June 2019 to November 2019. We are looking to find estimates for these missing values. Analysis were done using R Version 3.6.2 [1].

2 Methods

To find the missing values for OD oxygen and OD Naloxone, we propose the following approach. First, we apply a Generalized Linear Model (GLM) with a binomial distribution [2] to both available data for OD oxygen and OD Naloxone. Second, we use the resulting model to predict the missing values. The GLM model with binomial distribution writes as follows:

$$Y_i \sim B(n_i, \pi_i),$$

$$E(Y_i) = \pi_i n_i,$$

$$Var(Y_i) = n_i \pi_i (1 - \pi_i),$$

$$\pi_i = a_0 + a_1 t_i,$$

where Y_i is the number of times either oxygen or Naloxone was used, n_i is the number of overdoses, π_i is the probability that either oxygen or Naloxone was used, i is the index corresponding to the date. a_0 and a_1 are the unknown regression coefficients. Finally, $t_i = i$ corresponds to the number of months from the beginning of the study starting from $t_1 = 1$. Note that the missing values happen for t_{20} to t_{25}.

3 Results

In this section, we only present the results.

3.1 Oxygen

We detected overdispersion, and corrected the standard errors using a quasi- GLM model. Figure 1 shows the results, where the dots correspond to the available data, and the vertical lines are where the data is missing.

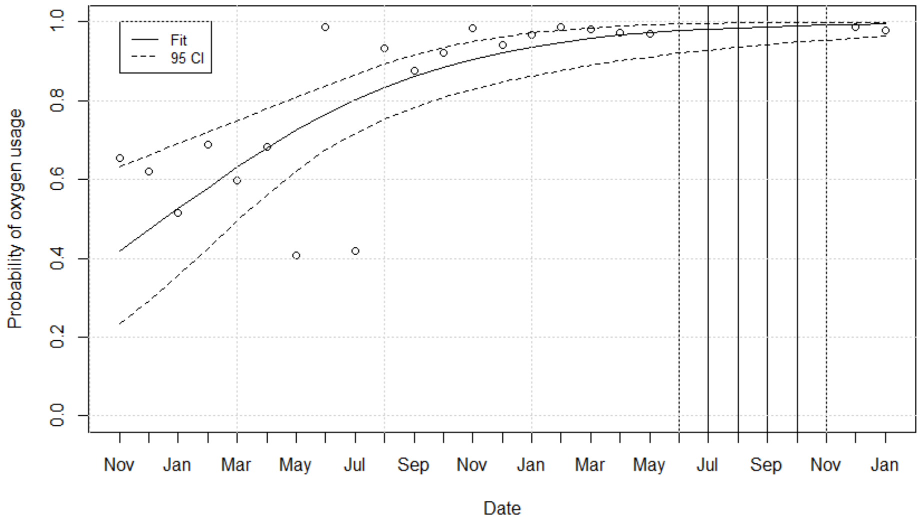

Figure 1: Probability of oxygen usage

Missing values for oxygen usage were derived by rounding the fitted values. Confidence intervals for missing values were derived by taking the floor and ceil functions from the 95% CI. Results are provided in Figure 2.

3.2 Naloxone

We detected overdispersion, and corrected the standard errors using a quasi- GLM model. Also, the explanatory variable t was found to be not significant, and therefore we set $a_1 = 0$, keeping only the intercept. Figure 3 shows the results, where the dots correspond to the available data, and the vertical lines are where the data is missing.

date	nOD	min_Oxygen	pred_Oxygen	max_Oxygen	min	predicted	max
2019-06-01	57	52.45	55.71	56.65	52	56	57
2019-07-01	90	83.56	88.35	89.60	83	88	90
2019-08-01	64	59.89	63.05	63.79	59	63	64
2019-09-01	39	36.76	38.53	38.91	36	39	39
2019-10-01	49	46.47	48.53	48.91	46	49	49
2019-11-01	73	69.62	72.43	72.91	69	72	73

Figure 2: Oxygen usage for missing months

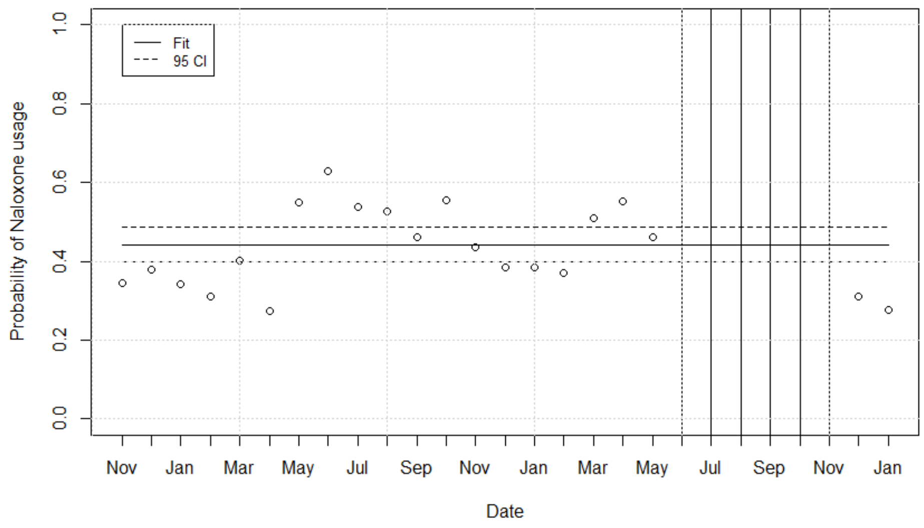

Figure 3: Probability of Naloxone usage

Missing values for Naloxone usage were derived by rounding the fitted values. Confidence intervals for missing values were derived by taking the floor and ceil functions from the 95% CI. Results are provided in Figure 4.

date	nOD	min_Naloxone	pred_Naloxone	max_Naloxone	min	predicted	max
2019-06-01	57	22.73	25.22	27.76	22	25	28
2019-07-01	90	35.89	39.82	43.83	35	40	44
2019-08-01	64	25.52	28.32	31.17	25	28	32
2019-09-01	39	15.55	17.26	18.99	15	17	19
2019-10-01	49	19.54	21.68	23.86	19	22	24
2019-11-01	73	29.11	32.30	35.55	29	32	36

Figure 4: Naloxone usage for missing months

4 Appendix

4.1 Oxygen

Summary plots for the GLM model are given in Figure 5.

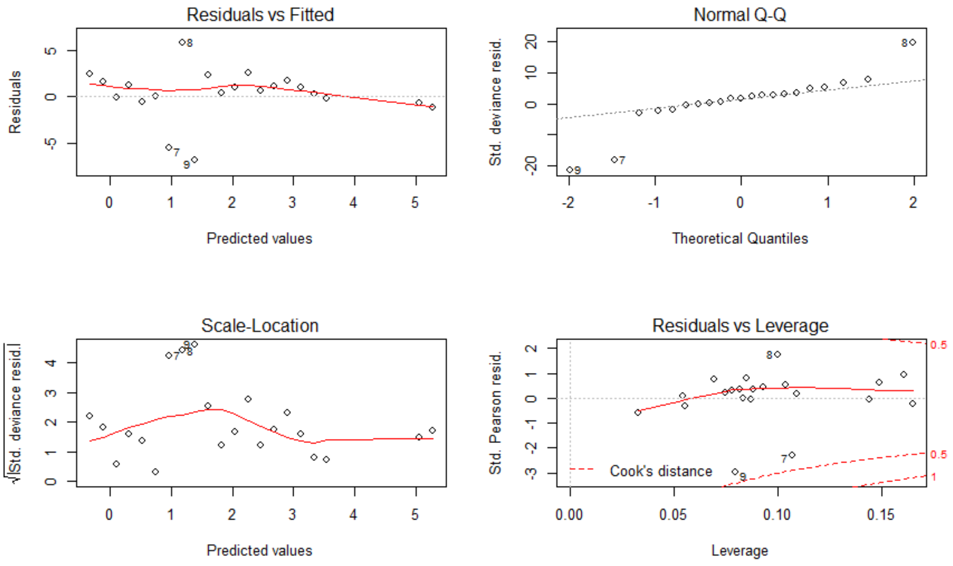

Figure 5: Summary plots: Quasi-binomial GLM for oxygen usage

4.2 Naloxone

Summary plots for the GLM model are given in Figure 6.

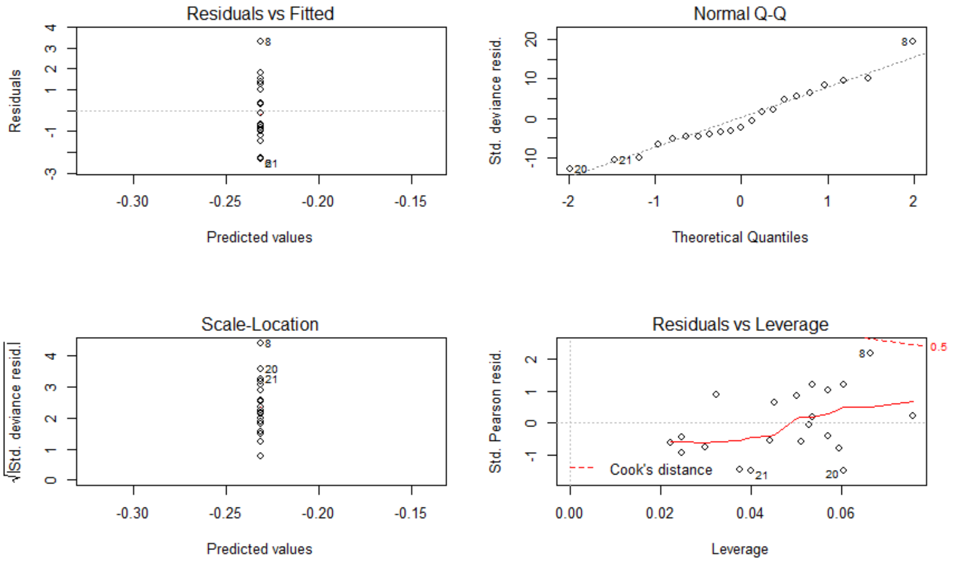

Figure 6: Summary plots: Quasi-binomial GLM for Naloxone usage

References

R Core Team. R: A language and environment for statistical computing. R Foundation for Statistical Computing, Vienna, Austria. 2013

Zuur, A., Ieno, E.N., Walker, N., Saveliev, A.A., and Smith, G.M. Mixed effects models and extensions in ecology with R. Springer, 2009
